# Analysis of the efficacy and safety of a combined gemcitabine, oxaliplatin and pegaspargase regimen for NK/T-cell lymphoma

**DOI:** 10.18632/oncotarget.8643

**Published:** 2016-04-07

**Authors:** Jing-hua Wang, Hua Wang, Yan-jun Wang, Zhong-jun Xia, Hui-qiang Huang, Wen-qi Jiang, Yue Lu

**Affiliations:** ^1^ Department of Hematological Oncology, Sun Yat-Sen University Cancer Center, Guangzhou, China; ^2^ State Key Laboratory of Oncology in South China, Guangzhou, China; ^3^ Collaborative Innovation Center for Cancer Medicine, Guangzhou, China; ^4^ Department of Urology, Sun Yat-Sen University Cancer Center, Guangzhou, China; ^5^ Department of Medical Oncology, Sun Yat-Sen University Cancer Center, Guangzhou, China

**Keywords:** extranodal NK/T-cell lymphoma, gemcitabine, oxaliplatin, pegaspargase, adverse effects

## Abstract

Extranodal natural killer/T-cell lymphoma (ENKTL) is an aggressive neoplasm with a poor outcome. Novel L-asparaginase-based treatment regimens, such as GELOX (gemcitabine, oxaliplatin, and L-asparaginase) and P-gemox (gemcitabine, oxaliplatin, and pegaspargase), have shown promising results against stage IE/IIE ENKTL. To define the general applicability of P-gemox, in a retrospective analysis we examined the efficacy and safety of P-gemox in a cohort of 117 patients with newly diagnosed or relapsed/refractory ENKTL. Treatment included 2 to 8 cycles of P-gemox: intravenous gemcitabine (1250 mg/m^2^) and oxaliplatin (85 mg/m^2^) and intramuscular pegaspargase (2500 IU/m^2^) on day 1 and repeated every 2 weeks, or intravenous gemcitabine (1000 mg/m^2^) on days 1 and 8 and intravenous oxaliplatin (130 mg/m^2^) and intramuscular pegaspargase (2500 IU/m^2^) on day 1 and repeated every 3 weeks. Upon completion of treatment, the overall response rate was 88.8%, and responses were similar for newly diagnosed and relapsed/refractory patients. After a median follow-up of 17 months, the 3-year overall and progression-free survival rates were 72.7% and 57.8%, respectively. Multivariate analysis showed that CR after treatment was the most significant factor affecting survival. P-gemox thus appears to be an effective and well-tolerated treatment for patients with ENKTL.

## INTRODUCTION

Although rare in the world, extranodal natural killer (NK)/T-cell lymphoma (ENKTL) is diagnosed comparatively frequently in Asia and Latin America and accounts for 5% to 10% of all malignant lymphomas in China [1, 2]. ENKTL most often originates in the nasal and upper aerodigestive tract, but can also arise in the skin, soft tissue, gastrointestinal tract, and testis [1, 3, 4]. For patients with localized ENKTL, radiation therapy (RT) alone yields overall response rates (ORRs) of 77% to 100%, with complete response (CR) rates of 52% to 100% [5–8]. On the other hand, the disease recurs in 25% to 40% of those patients [5], suggesting chemotherapy is needed to reduce the incidence of systemic failure. Patients with advanced-stage or relapsed/refractory ENKTL follow an extremely aggressive clinical course, during which chemotherapy is a mainstay treatment [9]. However, outcomes are most often poor with conventional CHOP-based chemotherapy regimens, as > 60% of patients suffer resistant disease [10–13]. The high incidence of resistance is likely related to the high concentrations of P-glycoprotein within ENKTL cells, which results in the tumor cells exhibiting multidrug resistance (MDR) [14, 15].

Several novel chemotherapy regimens based around L-asparaginase have recently emerged. For example combined dexamethasone, methotrexate, ifosfamide, L-asparaginase, and etoposide (SMILE) or combined asparaginase, methotrexate, and dexamethasone (AspaMetDex) achieve a complete response (CR) in 40% to 60% of cases [16–18]. The anticancer effect of L-asparaginase is not affected by MDR due to its unique mechanism. L-Asparaginase digests serum asparagine, and since ENKTL cells cannot synthesize asparagine themselves, tumor cell proliferation is suppressed [19]. PEG-asparaginase (pegaspargase), a form of *Escherichia coli* L-asparaginase covalently linked to polyethylene glycol, was rationally synthesized to decrease the immunogenicity of the enzyme and prolong its half-life. PEG-asparaginase has been granted approval as a first-line drug for the treatment of acute lymphoblastic leukemia [20] and also showed good therapeutic effect against ENKTL in recent clinical trials [21–23]. In a prospective study at our center, GELOX (gemcitabine, oxaliplatin, and L-asparaginase) and P-gemox (gemcitabine, oxaliplatin, and pegaspargase) regimens were tested in patients with stage IE/IIE ENKTL, and the results were promising [22]. To define the general applicability of P-gemox, in the present study, we systematically evaluated the potential efficacy and safety of this regimen in a large cohort of patients with newly diagnosed and relapsed/refractory ENKTL.

## RESULTS

### Patient characteristics

The patient characteristics are listed in Table 1. Our cohort included 96 newly diagnosed and 21 relapsed/refractory patients. The median age was 43 years (range, 13 years to 77 years), with 92 patients (78.6%) less than 55 years old. The male:female ratio was 2.2:1. The majority of patients (84.6%) were UENKTL cases - i.e., their ENKTLs orginated in the upper aerodigestive tract - and they showed good ECOG PS, IPI, and early-stage disease based on the Ann Arbor staging system. Eight patients with hepatitis B surface antigen (HBsAg)-positive, and were treated with antiviral medications. For relapsed/refractory patients, previous treatments included anthracycline-containing regimens (cyclophosphamide, doxorubicin, vincristine, and prednisolone [CHOP] or CHOP-like regimens) alone (*N* = 17) or followed by radiotherapy (*N* = 2), the 2/3DeVIC regimen (dexamethasone, etoposide, ifosfamide, and carboplatin) with radiotherapy (*N* = 1), and radiotherapy alone (*N* = 1).

**Table 1:** Characteristics of ENKTL patients treated using the P-gemox regimen

Characteristic	Newly diagnosed	Relapsed/refractory	Overall (%)
No. Age, years	96	21	117
<55	73	19	92(78.6)
≥55	23	2	25(21.4)
Gender			
Male	67	13	80(68.4)
Female	29	8	37(31.6)
Primary site			
UENKTL	81	18	99(84.6)
EUENKTL	15	3	18(15.4)
B symptoms			
Present	50	10	60(51.3)
Absent	46	11	57(48.7)
sLDH			
Normal	66	15	81(69.2)
Increased	30	6	36(30.8)
ECOG PS			
0	72	18	90(76.9)
1	19	3	22(18.8)
2	5	0	5(4.3)
Ann Arbor stage			
I/II	78	12	90(76.9)
III/IV	18	9	27(23.1)
IPI score			
≤1	70	12	82(70.1)
>1	26	9	35(29.9)

Abbreviations: P-gemox, gemcitabine,oxaliplatin and pegaspargase; UENKTL, upper aerodigestive tract NK/T-cell lymphoma; EUENKTL, upper aerodigestive tract NK/T-cell lymphoma; B symptoms include unexplained fever with temperature above 38C, night sweats or weight loss more than 10% within 6 months; sLDH, serum lactate dehydrogenase; ECOG PS, Eastern Cooperative Oncology Group performance status; IPI, International Prognostic Index.

### Treatment and response

The median number of P-gemox cycles among the 117 patients was 4 (range, 2-8 cycles). A total of 65 patients received radiotherapy, and the number of newly diagnosed patients who received radiotherapy was comparable to the number in relapsed/refractory patients. Other patients did not receive radiotherapy because of the high cost of treatment, disease progression, previous radiotherapy, or other personal reasons. Eight patients underwent hematopoietic stem cell transplantation (HSCT) after achieving interim CR or PR.

For the whole cohort, interim analysis showed a CR rate of 34.2% and an ORR rate of 80.3%. By treatment completion, the CR rate had improved to 58.9% and the ORR rate to 88.8%, whereas two patients remained SD, and seven developed PD. Treatment outcomes are summarized in Table 2; note that the interim and completion response rates were comparable between newly diagnosed and relapsed/refractory patients.

**Table 2:** Treatment outcomes to P-gemox chemotherapy

Disease status	*n*	Interim CR(%)	*p*	Interim ORR(%)	*p*	CompletionCR(%)	*p*	CompletionORR(%)	*p*
Newly diagnosed	96	32(33.3)	0.800	80(83.3)	0.125	58(60.4)	0.625	87(90.6)	0.247
Relapsed/refractory	21	8(38.1)		14(66.6)		11(52.4)		17(80.9)	
All patients	117	40(34.2)		94(80.3)		69(58.9)		104(88.8)	

Abbreviations: P-gemox, gemcitabine, oxaliplatin and Pegaspargase; CR, complete response; ORR, overall response rate.

### Survival

**Figure 1:**
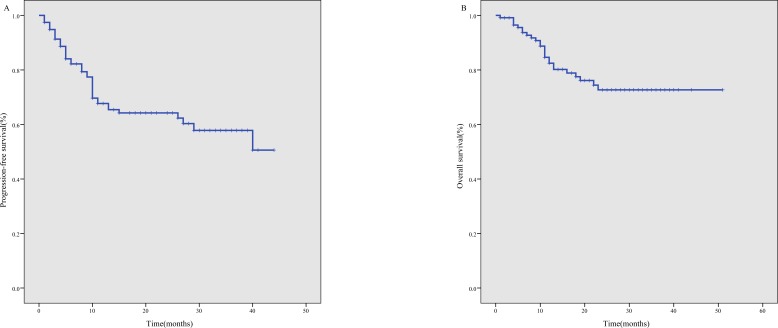
Survival curves for 117 ENKTL patients treated using the P-gemox regimen **A.** progression- free survival, **B.** overall survival.

The median follow-up time was 17 months, with a range of 1 month to 51 months. The 3-year PFS was 57.8% (95% CI, 47.2%-68.4%) for the whole cohort (Figure 1A), 65.4% (95% CI, 53.4%-77.4%) for newly diagnosed patients and 23.7% (95% CI, 4.6%-42.8%) for relapsed/refractory patients. The 3-year OS was 72.7% (95% CI, 63.4%-81.9%) for the whole cohort (Figure 1B), 75.6% (95% CI, 66.0%-85.3%) for newly diagnosed patients, and 57.7% (95% CI, 30.0%-85.4%) for relapsed/refractory patients.

**Figure 2:**
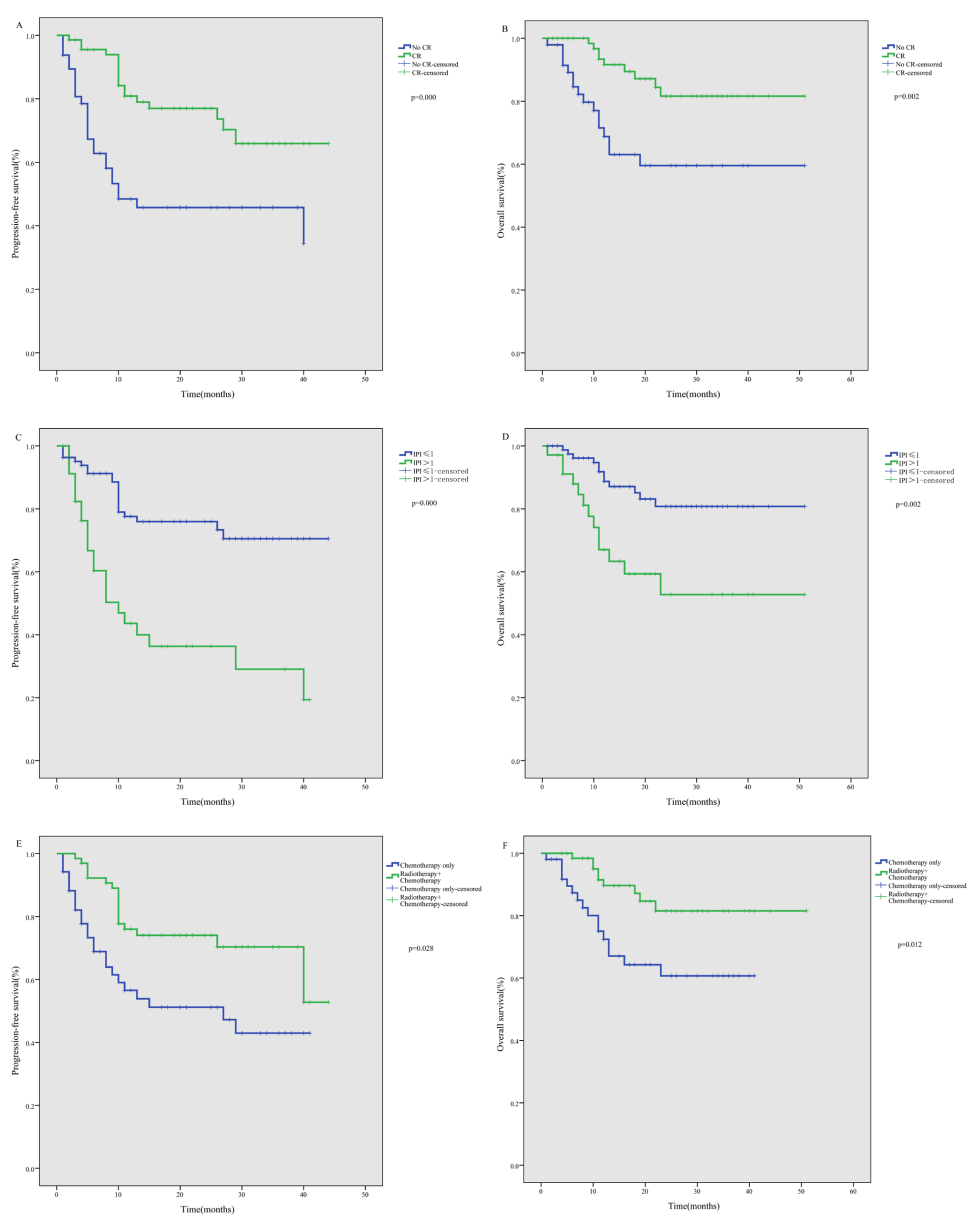
Subgroup analysis of survival among ENKTL patients treated using the P-gemox regimen **A.** Significant impact of treatment response on progression-free survival (PFS). **B.** Significant impact of treatment response on overall survival (OS). **C.** Significant impact of IPI on PFS. **D.** Significant impact of IPI on OS. **E.** Significant impact of radiotherapy on PFS among newly diagnosed patients. **F.** Significant impact of radiotherapy on OS among newly diagnosed patients.

In a subgroup analysis, newly diagnosed patients showed better PFS than relapsed/refractory patients (*P* < 0.001), but there was no significant difference in OS (*P* = 0.358). Similarly, PFS among stage I-II patients was better than among stage III-IV patients (*P* < 0.001), but again there was no significant difference in OS (*P* = 0.166). Our study also showed that both the PFS (*P* < 0.001) and OS (*P* = 0.002) rates were significantly better among patients who attained a CR by the end of treatment than among those without a CR (Figure 2A to 2B). In addition, a lower IPI score predicted a significantly better PFS (*P* < 0.001) and OS (*P* = 0.002) (Figure 2C to 2D). Finally, we found that newly diagnosed patients who received combined radiotherapy and chemotherapy showed better PFS (*P* = 0.028) and OS (*P* = 0.012) than those who received chemotherapy alone (Figure 2E-2F).

### Prognostic factors for PFS and OS

Univariate analysis of the whole cohort revealed that the significant factors affecting PFS included newly diagnosed or relapsed/refractory at entry (*P* < 0.001), EUENKTL (primary tumors at any site excluding nasal disease) (*P* = 0.047), stage (*P* < 0.001), IPI (*P* < 0.001), and CR status (*P* < 0.001). For newly diagnosed patients, EUENKTL (*P* = 0.032), B symptoms (*P* = 0.032), stage (*P* = 0.004), IPI (*P* < 0.001), and CR status (*P* = 0.001) were significant factors. Multivariate analysis showed that newly diagnosed or relapsed/refractory at entry (*P* = 0.004) and CR status (*P* = 0.013) remained significant independent factors for the whole cohort, while IPI (*P* = 0.005) and CR status (*P* = 0.021) significantly affected PFS for newly diagnosed patients (Table 3).

**Table 3:** Univariate and multivariate analysis of prognostic factors for survivals after P-gemox therapy

Clinical factors	Progression- free survival				Overall survival			
	Univariate		Multivariate		Univariate		Multivariate	
	*p*	HR (%95 CI)	*p*	HR (%95 CI)	*p*	HR (%95 CI)	*p*	HR (%95 CI)
Whole cohort								
Newly diagnosed or relapsed/refractory	0.000	3.362(1.782-6.341)	0.004	2.642(1.375-5.079)	0.361	0.654(0.261-1.638)	—	—
Age	0.564	0.805(0.385-1.685)	—	—	0.261	0.609(0.254-1.458)	—	—
EUENKTL	0.047	1.988(0.997-3.962)	—	—	0.030	2.457(1.060-5.696)	—	—
LDH	0.312	1.384(0.735-2.603)	—	—	0.231	1.623(0.729-3.614)	—	—
Stage	0.000	3.082(1.659-5.726)	—	—	0.169	1.790(0.772-4.150)	—	—
IPI	0.000	3.679(1.997-6.780)	—	—	0.002	3.163(1.441-6.940)	0.047	2.322(1.013-5.324)
CR after treatment	0.000	2.970(1.597-5.520)	0.013	2.265(1.191-4.309)	0.002	3.376(1.489-7.654)	0.027	2.619(1.117-6.139)
Newly diagnosed								
EUENKTL	0.032	2.410(1.052-5.517)	—	—	0.009	3.222(1.265-8.208)	—	—
Age	0.493	0.740(0.313-1.753)	—	—	0.569	0.744(0.268-2.068)	—	—
B symptoms	0.032	2.400(1.050-5.484)	—	—	0.291	1.645(0.647-4.182)	—	—
LDH	0.074	1.977(0.922-4.239)	—	—	0.097	2.110(0.857-5.195)	—	—
Stage	0.004	3.025(1.377-6.643)	—	—	0.028	2.725(1.072-6.927)	—	—
IPI	0.000	4.791(2.221-10.337)	0.005	4.421(1.552-12.593)	0.000	4.913(1.967-12.268)	0.003	6.450(1.872-22.228)
CR after treatment	0.001	3.502(1.596-7.683)	0.021	2.808(1.170-6.740)	0.000	5.546(1.994-15.431)	0.001	4.238(1.387-12.952)

Abbreviations: EUENKTL, extra-upper aerodigestive tract NK/T-cell lymphoma; IPI, International Prognostic Index; B symptoms include unexplained fever with temperature above 38°C, night sweats or weight loss more than 10% within 6 months; HR, hazard ratio; CI, confidence interval.

Univariate analysis of the whole cohort revealed that the significant factors affecting OS included EUENKTL (*P* = 0.030), IPI (*P* = 0.002) and CR status (*P* = 0.002). For newly diagnosed patients, EUENKTL (*P* = 0.009), stage (*P* = 0.028), IPI (*P* < 0.001), and CR status (*P* < 0.001) were significant predictors. Multivariate analysis showed that IPI (*P* = 0.047 and 0.003) and CR status (*P* = 0.027 and 0.001) remained significant independent factors for the whole cohort and for newly diagnosed patients (Table 3).

### Adverse effects

Treatment-related adverse effects are shown in Table 4. The most common adverse effect of the P-gemox regimen was hematological toxicity. However, non-hematologic toxicities were also frequent, though most reported cases were mild. Gastrointestinal disorders, including nausea, emesis, and diarrhea, were observed in 40 patients. Mild coagulation abnormalities, which were reflected by prolongation of the activated partial thromboplastin time (APTT), were observed in 54 patients. Two patients developed left upper limb deep-vein thrombosis, but revascularization was achieved following treatment with anticoagulant. One patient developed an allergic reaction, and two patients developed pancreatitis. Because of adverse effects, the doses administered to 11 patients were reduced or chemotherapy was delayed. A 72-year-old man died from suffocation during the second cycle of chemotherapy. No difference in the incidence of adverse effects was observed between newly diagnosed and relapsed/refractory patients, except for hyperbilirubinaemia (*P* < 0.001) and hypofibrinogenemia (*P* = 0.03).

**Table 4:** Adverse effects of P-gemox chemotherapy

Adverse effects	Newly diagnosed	Relapsed/refractory	All cases(%)	*p*
Leukopenia				
Grade 1/2	55	11	66(56)	
Grade 3/4	18	8	26(22)	0.08
Neutropenia				
Grade 1/2	45	9	54(46)	
Grade 3/4	28	7	35(30)	0.79
Anemia				
Grade 1/2	68	19	87(74)	
Grade 3/4	18	2	20(17)	0.52
Thrombocytopenia				
Grade 1/2	20	5	25(21)	
Grade 3/4	17	4	21(18)	1.00
Gastrointestinal disorders	34	6	40(34)	0.62
Increased transaminases				
Grade 1/2	72	17	89(76)	
Grade 3/4	10	0	10(8.5)	0.21
Hyperbilirubinaemia	46	2	48(41)	0.00
Coagulopathy	47	7	54(46)	0.23
Hypofibrinogenemia	50	5	55(47)	0.03
Increase in BUN	29	2	30(26)	0.06
Hypertriglyceridemia	61	14	75(64)	1.00
Hyperglycemia	23	5	28(24)	1.00
Hypoglycemia	24	5	29(25)	1.00
Hypoalbuminemia	50	5	55(47)	1.00
deep-vein thrombosis	1	1	2(1.7)	0.33
allergic reactions	1	0	1(0.8)	**1.00**
pancreatitis	2	0	2(1.7)	**1.00**

Abbreviations: P-gemox, gemcitabine, oxaliplatin and Pegaspargase.

## DISCUSSION

ENKTL is a highly invasive tumor with a short doubling time and poor prognosis. How best to treat ENKTL has been a troublesome issue because the disease frequently shows resistance to anthracycline-based chemotherapy due to MDR, and local radiation therapy often fails to prevent systemic disease relapse or progression [5]. With this study, we aimed to determine a treatment strategy for ENKTL that is more effective and better tolerated than previously reported treatments.

Gemcitabine is a nucleoside analogue that has shown promising results in non-Hodgkin lymphoma [24–26]. A retrospective study of gemcitabine alone or as part of a chemotherapy regimen for relapsed/refractory ENKTL recently reported that 40% of patients showed an objective response [27]. Oxaliplatin is another drug effective against non-Hodgkin lymphoma, with a single-drug objective response rate of 27% to 40% [28]. When gemcitabine and oxaliplatin were used in combination, a synergistic effect was observed *in vitro* and in clinical studies with lymphoma [29, 30]. L-asparaginase is an antitumor drug with a unique mechanism of action, and several L-asparaginase-based regimens have exhibited promising results in patients with newly diagnosed or relapsed/refractory ENKTL. Pegaspargase, the apegylated form of L-asparaginase, is associated with a lower incidence of induction of anti-asparaginase antibodies and exhibits more prolonged asparaginase activity than native asparaginase [31]. Results from several case reports and clinical studies with small sample sizes suggest pegaspargase may be effective in selected patients [21, 22, 32, 33]. We selected pegaspargase for its ability to induce more prolonged continuous asparagine depletion as well as for its ease of administration, as only a single treatment every 2 to 3 weeks is required. We then developed the P-gemox regimen, which includes a combination of gemcitabine, oxaliplatin, and pegaspargase, all of which are non-anthracycline drugs that exhibited significant activity and non-overlapping toxicity in previous studies, and are unaffected by MDR.

In the present study, the ORR of P-gemox for the whole cohort was 80.3% at interim and 88.8% on completion, and 53 (45.3%) patients were alive in continuous CR at the end of follow-up. This highlights the favorable long-term outcome of this regimen and demonstrates that P-gemox is an effective regimen that induces a swift response. Moreover, the response rates were similar for newly diagnosed patients receiving P-gemox as upfront therapy and relapsed/refractory patients receiving it as salvage.

It is noteworthy that whereas the survival analysis indicated that newly diagnosed patients showed better PFS than relapsed/refractory patients, there was no significant difference in OS. We think there are two reasons for this. First, the number of relapsed/refractory patients included in the present study is relatively small, so the conclusion remains to be confirmed in larger, multiple-center trials. Second, when disease progression began again in relapsed/refractory patients after treatment with the P-gemox regimen, they were treated with HSCT or new drugs such as Lenalidomide, which enabled most to achieve remission and survive. Nonetheless, we suggest newly diagnosed patients will show better OS than relapsed/refractory patients if follow-up is extended. In addition, among newly diagnosed patients, those who received combined radiotherapy and chemotherapy showed better PFS and OS than those who received chemotherapy only. This indicates that radiotherapy serves an important purpose in the treatment of newly diagnosed ENKTL.

For comparison of our results with those of earlier studies of ENKTL, Table 5 summarizes the latest published results obtained with L-asparaginase-based regimens. These regimens all yielded promising results in patients with newly diagnosed or relapsed/refractory ENKTL. Table 5 shows that the treatment response and survival outcomes achieved with the P-gemox regimen are superior or similar to those obtained with other regimens, which is indicative of the excellent antitumor effect of the P-gemox regimen.

**Table 5:** Study comparison of pegaspargase- or L-asparaginase-based regimens in the treatment of ENKTL

			Response		Survival		Adverse effects		
Disease status	No.	Treatment	ORR	CR	OS	PFS	Grade 3/4 neutropenia	Grade 3/4 hepatotoxicity	Reference
Newly diagnosed, relapsed/refractory,any stage	117	P-gemox± sandwiched RT (56 Gy)	88%	59%	3 y: 73%	3 y: 58%	30%	8.5%	this study
Newly diagnosed, relapsed/refractory, any stage	87	SMILE± sandwiched RT (50 Gy)	81%	66%	5 y: 50%	4 y DFS: 64%	67%	7%	17
Newly diagnosed, stage IV, or relapsed/refractory	38	SMILE	79%	45%	1 y: 55%	1 y: 53%	100%	32%	16
Relapsed/refractory	19	AspaMetDex	78%	61%	2 y: 40%	2 y: 40%	42%	16%	18
Newly diagnosed, stage I/II nasal	27	GELOX ±sandwiched RT (56 Gy)	96%	74%	2 y: 86%	2 y: 86%	33.3%	3.7%	22

Abbreviations: No., number; ORR, overall response rate; CR, complete remission; OS, overall survival; PFS, progression-free survival; DFS, disease-free survival; RT, radiotherapy; y, year; P-gemox, gemcitabine,oxaliplatin and pegaspargase; SMILE, methotrexate, ifosfamide, dexamethasone, etoposide, and L-asparaginase; AspaMetDex, L-asparaginase, methotrexate, and dexamethasone; GELOX, gemcitabine, oxaliplatin, and L-asparaginase.

Multivariate analysis of the whole cohort showed that relapsed/refractory at entry, EUENKTL, stage III-IV, IPI > 1, and no CR were independent factors associated with relapse or poor survival in ENKTL. These factors differed from those reported by Lee et al., which included B symptoms, stage, LDH level, and lymph node involvement as independent prognostic factors [3]. This difference may be due to the smaller number of participants in our study or the high efficacy of pegaspargase. Investigations using a larger patient cohort will be needed to identify the appropriate prognostic factors for ENKTL in the era of non-anthracycline-based chemotherapy.

Among adverse events, grade 3/4 toxicities were mainly hematological. Dose reduction and timely application of G-CSF were essential when patients experienced severe cytopenias during or after chemotherapy. Other support treatments, such as platelet transfusion and infection prevention, were also necessary. Commonly reported side effects of pegaspargase therapy include gastrointestinal disorders, liver dysfunction, coagulopathy, hypertriglyceridemia, hyperglycemia, and hypoalbuminemia [34]. In our study, grade 1 and 2 toxicities were frequently relating to these side effects and could be well controlled by symptomatic treatments. Venous thrombosis is another known adverse event associated with asparaginase. During our study period, two patients developed left upper limb deep venous thrombosis, but revascularization was achieved following anticoagulant treatment. Thus, patients should be monitored closely for thrombosis during follow-up and in subsequent trials. One allergic reaction and two pancreatitis cases were also observed in this study, and one treatment-related death was attributed to suffocation. The hematologic toxicities and liver dysfunction seen in our study were considerably milder than those reported with the SMILE and AspaMetDex regimens (Table 5). In sum, the P-gemox regimen presented acceptable toxicity profiles.

In conclusion, this study demonstrated that P-gemox is a more effective and well-tolerated treatment for patients with newly diagnosed, relapsed, or refractory ENKTL.

## MATERIALS AND METHODS

### Patients

From October 2010 to January 2015, a total of 117 patients with newly-diagnosed or relapsed/refractory ENKTL who were treated with P-gemox at Sun Yat-sen University Cancer Center were identified and included in this retrospective analysis. Clinical information was obtained through a review of medical records. The inclusion criteria for this retrospective study were (1) a pathologically confirmed diagnosis of ENKTL based on the WHO classification of Tumors of Hematopoietic and Lymphoid Tissues; (2) NK/T-cell type proven by immunohistochemistry, Epstein-Barr virus (EBV) *in situ* hybridization or flow cytometry; (3) adequate hematologic function (hemoglobin > 90 g/L, absolute neutrophil count > 1.5×10^9^/L, and platelet count > 100×10^9^/L), renal function (serum creatinine ≤ 1.5 mg/dL and creatinine clearance ≥ 50 mL/min), and hepatic function (total bilirubin ≤ 2 times the upper limit of normal and aspartate and alanine transaminase levels ≤ 3 times the upper limit of normal); (4) no previous or concomitant malignancies; and (5) a complete set of clinical information and follow-up data. Patients with aggressive NK-cell leukemia, negative EBV *in situ* hybridization, CNS involvement, pregnancy, or lactation were excluded. Based on the primary tumor site, the cancers were classified as upper aerodigestive tract NK/T-cell lymphoma (UENKTL: primary tumors confined to the nasal cavity, nasopharynx, paranasal sinuses, tonsils, hypopharynx, and larynx) or extra-UENKTL (EUENKTL: primary tumors at all sites excluding nasal disease) [3]. Primary tumors within the nasal cavity with secondary spread to other organs were regarded as UENKTL. This study design was approved by the SunYat-sen University Cancer Center Research Ethics Board. All of the patients agreed that their medical information could be used for medical research, and signed an informed consent during their first visit.

Patients were staged on the basis of Ann Arbor staging system; International Prognostic Index (IPI) scores, which were calculated on the basis of demographic characteristics; a physical examination; Eastern Cooperative Oncology Group performance status (ECOG PS); systemic B symptoms (unexplained fever with temperature above 38°C, night sweats or weight loss exceeding 10% within 6 months); complete blood count; serum biochemistry with lactate dehydrogenase (LDH); computed tomography (CT) scans of the chest, abdomen, and pelvis; magnetic resonance imaging (MRI) of the head and neck; and bilateral bone marrow aspiration or biopsy. Positron emission tomography (PET)-CT scans, bone marrow Epstein-Barr virus-encoded small RNA (EBER) staining, and Epstein-Barr virus (EBV) DNA blood levels were not included in the routine staging investigations in the study. Follow-up information was obtained from the patients' medical records or by telephone.

### Treatment

The P-gemox regimen entailed intravenous injection of gemcitabine (1250 mg/m^2^) and oxaliplatin (85 mg/m^2^) and intramuscular injection of pegaspargase (2500 IU/m^2^) on day 1, repeated every 2 weeks. Alternatively, the regimen entailed intravenous injection of gemcitabine (1000 mg/m^2^) on days 1 and 8 and oxaliplatin (130 mg/m^2^) on day 1 with intramuscular injection of pegaspargase (2500 IU/m^2^) on day 1, repeated every 3 weeks. The two regimens used similar dose intensities (i.e., the dose of effective drug administered per unit of time [in mg/m^2^ per week]). For patients who were in poor condition or experienced severe toxicity, we reduced the doses as follows. For patients who experienced severe hematologic toxicity, all the drugs were reduced 25% in the next course. For patients who experienced severe gastrointestinal disorder, oxaliplatin was reduced 25% in the next course. For patients who experienced severe liver dysfunction or coagulopathy, pegaspargase was reduced 25% in the next course.

During the chemotherapy intervals, patients with hematologic toxicity and a white blood cell count < 2.0×10^9^/L or absolute neutrophil count ≤ 1.0×10^9^/L received granulocyte-colony-stimulating factor (G-CSF) (5 μg/kg/day, hypodermically injected) until the neutrophil counts recovered. Patients with localized ENKTL were referred for primary involved-field radiation (IFRT) after receiving a maximum of 6-8 initial chemotherapy cycles. If patients with localized ENKTL only achieved stable disease (SD) following two cycles, they were administered primary IFRT. If patients had not achieved CR after four cycles, they were given IFRT. For patients with advanced-stage or relapsed/refractory disease who had never been irradiated, IFRT of the primary anatomic site or residue lesion was administered after all chemotherapy treatments were complete. The IFRT median total dose was 56 Gy, with a range of 40 Gy to 60 Gy. In addition, patients could receive HSCT after achieving interim CR or PR. The decision was made at the discretion of the treating physicians, mainly on the basis of the patient's age and condition.

### Response and toxicity criteria

Treatment responses were assessed every two chemotherapy cycles and classified as CR, PR, SD, or PD according to the Revised Response Criteria for Lymphoma [35]. The overall response rate (ORR) was defined as the proportion of patients who achieved CR or PR. The interim response was assessed after two courses, and the completion response was defined as the response after the last course of P-gemox. After treatment was completed, patients were followed up and assessed by their oncologist in the outpatient department. Each assessment consisted of a physical examination, complete blood count, serum biochemistry (including LDH level), and either a CT scan, MRI of the involved region, or PET-CT scan. Toxicity after chemotherapy was evaluated according to the National Cancer Institute Common Terminology Criteria of Adverse Events v3.0.

### Statistical analysis

OS was calculated from the date of the first administration of P-gemox to the date of death or last follow-up. Progression-free survival (PFS) was measured from the date of the first administration of P-gemox to the date of disease progression, relapse or death from any cause. The χ^2^ test was used to calculate statistical group comparisons of categorical variables. Survival analysis was performed using the Kaplan-Meier method, and curves were compared using the log-rank test. Univariate and multivariate analyses of independent factors for survival were performed using the Cox proportional hazard model. Two-tailed *P* values of less than 0.05 were considered significant. Statistical analyses were performed by using SPSS 17.0 software.
